# 1186. Increased Respiratory Syncytial Virus (RSV) Viral Replication Leads to Increased Cytokine Production and Polarized Interferon Response in Infant Mucosal Epithelium

**DOI:** 10.1093/ofid/ofab466.1378

**Published:** 2021-12-04

**Authors:** Rebecca M Glowinski, Ki Wook Yun, Asuncion Mejias, Octavio Ramilo

**Affiliations:** 1 Nationwide Children’s Hospital, Columbus, Ohio; 2 Seoul National University College of medicine, Seoul

## Abstract

**Background:**

RSV is the most frequent etiology of pediatric lower respiratory tract infection. Most children hospitalized for RSV are previously healthy without known risk factors. Children with mild disease managed as outpatients (OP) have higher viral loads than those hospitalized with severe disease. OP children have higher concentrations of the mucosal interferon (IFN), IFNλ2/3, and IP-10, but no differences in IFNλ1. We examined how RSV replication impacts cytokine production kinetics in the nasal mucosa.

**Methods:**

Primary infant human nasal epithelial (iHNE) cells were collected from nasopharyngeal swabs and cultured on an air-liquid interface. Cultures were infected with 0.1 or 0.001 multiplicity of infection (MOI) of RSV-A, or mock infected. Concentrations of IFN-related (IFNα2, β, γ, λ1, λ2/3, and IP-10) and inflammatory (IL-1β, -6, -12, and TNFα) cytokines secreted to the apical and basolateral surfaces were quantified via immunoassay. Kinetics according to viral inocula were compared by ANOVA with Dunn post-hoc testing of the area under the curve (AUC) for each cytokine. Peak concentrations were compared according to MOI and secretion surface by 2-way ANOVA.

**Results:**

AUC of IFNs in both surfaces of RSV infected cells were significantly higher than those of mock infected. The 0.1 MOI RSV inoculum resulted in significantly higher AUCs for all IFN cytokines on both surfaces than the 0.001 MOI. Peak IFNλ1 concentrations were higher on the apical than basolateral side; peak IFNλ2/3 concentrations were higher on the basolateral side than apical. AUCs of inflammatory cytokines in RSV infection were significantly higher on the basolateral, but not apical, surfaces than mock; all basolateral inflammatory cytokines were higher in the 0.1 MOI than the 0.001 MOI.

**Conclusion:**

Higher RSV inoculum induces higher concentrations of IFN-related cytokines on both sides of epithelial cells, and higher concentrations of inflammatory cytokines on the basolateral side. Differential secretion of IFNλ1 and IFNλ2/3 to the apical and basolateral surfaces suggests they may play different roles in immune response during RSV infection. These data support viral replication as an important factor influencing RSV pathogenesis and severity through cytokine production.

**Disclosures:**

**Asuncion Mejias, MD, PhD, MsCS**, **Janssen** (Grant/Research Support, Advisor or Review Panel member)**Merck** (Grant/Research Support, Advisor or Review Panel member)**Roche** (Advisor or Review Panel member)**Sanofi** (Advisor or Review Panel member) **Octavio Ramilo, MD**, **Adagio** (Consultant)**Bill & Melinda Gates Foundation** (Grant/Research Support)**Janssen** (Grant/Research Support)**Lilly** (Consultant)**Merck** (Consultant, Grant/Research Support)**NIH** (Grant/Research Support)**Pfizer** (Consultant)**SANOFI** (Board Member)

